# A Physical Model-Based Observer Framework for Nonlinear Constrained State Estimation Applied to Battery State Estimation

**DOI:** 10.3390/s19204402

**Published:** 2019-10-11

**Authors:** Jonathan Brembeck

**Affiliations:** Institute of System Dynamics and Control, Robotics and Mechatronics Center, German Aerospace Center (DLR), 82234 Weßling, Germany; jonathan.brembeck@dlr.de; Tel.: +49-8153-28-2472

**Keywords:** nonlinear observer, kalman filter, constrained estimation, state of charge estimation, lithium-ion cell, hybrid simulation, functional mockup interface, modelica, AUTOSAR

## Abstract

Future electrified autonomous vehicles demand higly accurate knowledge of their system states to guarantee a high-fidelity and reliable control. This constitutes a challenging task—firstly, due to rising complexity and operational safeness, and secondly, due to the need for embedded service oriented architecture which demands a continuous development of new functionalities. Based on this, a novel model based Kalman filter framework is outlined in this publication, which enables the automatic incorporation of multiphysical Modelica models into discrete-time estimation algorithms. Additionally, these estimation algorithms are extended with nonlinear inequality constraint handling functionalities. The proposed framework is applied to a constrained nonlinear state of charge lithium-ion cell observer and is validated with experimental data.

## 1. Introduction

The robotic electric research plattfom DLR ROboMObil [1] enables many highly demanding control techniques, for example, for a parametric path following control or nonlinear optimization based control allocation (e.g., [2]). For implemtation and testing in a real-world experiment, all these approaches require precise and reliable knowledge of vehicle system states. Some of these states cannot be sensored directly, since an adequate sensor principle for the searched quantity is not available (e.g., determination of the state of charge (SOC) of a battery) or the sensor is expensive and therefore it is desirable to economize it (e.g., vehicle over ground velocity). The goal of this research is to develop a complete toolchain to assist the control systems engineer in the development of complex, continuous-time formulated prediction models for estimation purposes. These should be applied to discrete-time state estimation algorithms and the automatically generated code should afterwards be easily downloadable to an embedded microcontroller target. In this work, a novel framework for state estimation using detailed multiphysical continuous-time models designed in Modelica [3] is developed. It employs an intelligent separation of the model (encapsulated in a standardized FMI (Functional Mockup Interface) 2.0 for co-simulation [4,5]) and the estimation algorithm by utilizing modern computer technologies and recent developments in the Modelica language, which enable automated discretization, integration and derivative calculation of an object oriented, equation based prediction model. The Functional Mock-up Interface (or FMI) defines a standardized interface to be used in computer simulations to develop complex cyber-physical systems. The vision of FMI is to support this approach—if the real product is to be assembled from a wide range of parts interacting in complex ways, each controlled by a complex set of physical laws, then it should be possible to create a virtual product that can be assembled from a set of models that each represents a combination of parts, each a model of the physical laws as well as a model of the control systems (using electronics, hydraulics, digital software, etc.) assembled digitally [6]. Functional Mock-up Interface (FMI) is a tool independent standard to support both model exchange and co-simulation of dynamic models using a combination of XML-files and (compiled) C-code. FMI is supported by over 108 tools and is used by automotive, aerospace, robotics, and so forth, organizations throughout Europe, Asia and North America [3].

Beyond this, a direct usage of the state estimator on a cross platform embedded microcontroller target is also enabled.

### 1.1. State of the Art in Estimation Toolboxes Using Kalman Filter Techniques

The textbooks [7,8,9] are excellent starting points for the theoretical background on Kalman filtering and optimal state estimation. For this reason, only the most important steps of discrete nonlinear estimation and their interface to the estimation framework are given in Chapter 2 to guarantee readability and comprehension.

To the best of the author’s knowledge, the MATLAB toolbox EKF/UKF [10] and the ACADO toolkit [11] can be seen as the most advanced publicly available toolboxes for state estimation problems. Both require an analytically derived, by hand or via symbolic preprocessing, (time-discrete) prediction model provided by the user. This is nontrivial, error-prone and time-consuming to utilize for a concrete estimation application with a complex nonlinear model. In contrast, a Modelica [3] simulator, for example, Dymola, is able to analyze the structure of an acausal interconnected multiphysical model, to inline the discretization method and finally to transform it to an order reduced ordinary differential equation representation with analytic derivative calculation features. Another issue is that, in the case of the first toolbox [10], no functionality is provided to directly cross compile, that is, compile for an execution system different to that of the development system and download the result to a real-time target.

In this work, a formulation and code generation framework is proposed that makes use of an estimation-problems-specific, extended FMU (Funtional Mockup Unit) 2.0 co-simulation interface [4], providing the possibility of formulating prediction model equations in continuous-time by means of the Modelica language. In addition to the implementation of the well-proven Kalman filter algorithms (e.g., in [8]), fast and reliable theory extensions for nonlinear constraint handling are given in Chapter 3.

In [12] an approach is given that utilizes the older FMI 1.0 standard [4], which is not able to directly handle inline integration or model state events. This is similar to the approach in [13]; besides that, the authors of [12] use Python with PyFMI and not a Modelica environment, which facilitates the integration of the observer in combination with model based nonlinear inverse controllers or the Modelica Synchronous Control System Library in a single development environment [3].

### 1.2. State of the Art in Battery Modelling and State Estimation

The applied FMI-based framework estimates the state of charge in batteries of electric vehicles. The current state-of-art SOC estimation algorithms can be mainly classified into three categories [14]—model-based methods, algorithms relying on a battery’s physical model and data-driven approaches which assume the cell is a black box (not discussed here); cf. [15] for details. The interested reader can find a comprehensive overview of recent SOC estimation methods in [14]. Regarding the category of model-based methods, many research studies approach the SOC estimation problem using electrical circuit models for the lithium-ion cell dynamics in combination with (mainly) Kalman filter based estimation algorithms in different degrees of detail. The cell models based on equivalent circuit models are usually composed of one or two [16,17,18,19] resistor capacitor (RC) elements in parallel configuration, an additional resistor (R) in serial configuration and an SOC dependent open cell voltage (OCV) source. Their parameters and characteristics at different cell operation temperatures are fitted using optimization methods like recursive least squares [17] or multi-swarm particle optimization [20]. Moreover, promising results have been recently achieved, estimating the remaining lifespan of a li-ion cell using a novel particle learning algorithm in combination with a non-physical motivated exponential model [21]. Similar achievements with a dual-scale particle filter for predicting the cell’s remaining amount of energy including a comprehensive assessment of five equivalent circuit cell prediction models are given in [15]. Details on the effects of cell degradation and its modelling over its life-time can be found, for example, in [22,23].

The constrained nonlinear battery observer application shown here emphasizes the constrained nonlinear estimation framework technology and shows its versatility and impact for current research on embedded estimation technology, without having the ambition to outperform the most recent achievements in SOC estimation. In this publication, according to the classification in [14], a model based estimation method is exemplified for the usage with the physical model-based observer framework. The estimation method, a modified and improved version of [24,25], is based on an equivalent circuit model using a recursive (Kalman Filter) approach.

### 1.3. Contributions and Focus of the Work

This work presents a new highly automated framework for generating model based observers based on different types of Kalman filters extended with constraint handling algorithms for the use in embedded application which are explained in Chapter 2 and 3. The main contributions are the structutured approach from designing an object oriented acausal multi-physical model in the Modelica [3] language over to an automated equation sorting and a discretization process towards a ready to use nonlinear (constrained) Kalman filter that can be exported to a cross-platform target, for example, an AUTOSAR embedded controller. In Chapter 4, this approach is used to implement a lithium-ion battery state of charge (SOC) estimator application. Here, it can be highlighted that through the acausal formulation of the battery cell prediction model in continuous-time, it is easily possible to model the system in a hybrid combination of cell characteristic tables and state equations. In this way, it is possible to make various improvements and modifications to the cell prediction model while being able to automatically generate a discrete-time nonlinear observer algorithm. This provides a large benefit in comparison to the error-prone modelling approach with manual transformation from continuous to discrete-time (e.g., see [24,25]); it also enables a quick exchange of system models with different complexity, as discussed in [14,16,17,18,19]. The further extension of the model-based observer framework with a moving horizon estimation approach for compensating delayed measurements and improving the estimation, as well as its practical application to a state of the art vehicle position estimation problem formulation, is discussed in [26]. This article is an extended and improved version of these publications [13,27,28].

## 2. Design of a Modelica Based Estimation Framework

In this chapter, a method is developed to automatically generate nonlinear state estimators based on continuous-time Modelica models. The approach is based on an extended FMI 2.0 co-simulation interface [4] that interacts with the state estimation algorithms implemented in the DLR Kalman Filter Library [27].

With the raise of computational power in the last decades the possibility of implementing complex control strategies in real world applications has been enhanced tremendously. For most of them a good knowledge of the actual states is necessary. Often these are not directly measurable due to cost limitations or missing sensors. In the ITEA project MODRIO, one aim has been to develop state estimation technologies for plants that use the knowledge of complex models of the controlled system itself. These models are often designed, parameterized and optimized as multi-domain models in Modelica [3]. To reuse these models for estimation and control purposes, the FMI [4] turns out to be very helpful, since currently in Modelica it is not possible to directly call (e.g., integrate or state-manipulate) a model within a model.

In the Chapters 2.2 and 2.3 a brief introduction to the most important estimation algorithms, the extended Kalman filter and unscented Kalman filter, is given and the steps are identified where the model evaluations are necessary by means of the FMI. For time-discrete Kalman filter theory details, derivative-free methods and a numerical robust filter design using matrix decomposition and propagation, please inspect the appendix Chapter A in reference [2].

### 2.1. Model Evaluations in State Estimation Algorithms

For many control system tasks the plant model to be used in state estimation is naturally described as a nonlinear continuous-time state-space system:

(1) $$\begin{array}{c} \dot{x}=f\left(x,u\right),\\ \;y=h\left(x\right),\\ \;\;u\left(t\right)\in {\mathbb{R}}^{n_{u}},\;x\left(t\right)\in {\mathbb{R}}^{n_{x}},\;y\left(t\right)\in {\mathbb{R}}^{n_{y}},t\in \mathbb{R} \end{array}$$

where t is the time, u(t) is the vector of inputs,  x(t) is the vector of states and y(t) is the vector of outputs. Besides of manual, time-consuming and error-prone derivation of this representation, in this work the plant models are defined in Modelica and exported as a functional mockup unit (FMU) [4,5] using the Modelica simulator Dymola. Note that all the research results presented here are also valid if FMUs are generated by other tools and/or non-Modelica environments, as long as the FMU supports the extended FMI 2.0 co-simulation interface according to Table 1.

In a sampled data system (e.g. a microcontroller) the continuous-time model representation in Equation (1) cannot be used directly. Instead a time-discrete representation is needed and therefore the time-discrete transformation of Equation (1) with additive Gaussian noise is used in the sequel:

(2) $$\begin{array}{c} x_{k}\;=\;\;f_{k|k-1}\left(x_{k-1},u_{k-1}\right)+w_{k-1},\\ \;y_{k}\;=\;\;h\left(x_{k}\right)+v_{k},\\ \;\;w_{k}\;\sim \;N\left(0,Q_{k}\right),\\ \;\;v_{k}\;\sim \;N\left(0,R_{k}\right). \end{array}$$

here $t_{k}$ is the k -th sample time instant of a periodically sampled data system. We define $u_{k}\;=\;u\left(t_{k}\right)\;$, $x_{k}\;=\;x\left(t_{k}\right)$ and $y_{k}\;=\;y\left(t_{k}\right)\;$. The vectors $w_{k}$ and $v_{k}$ represent zero biased Gaussian white noise. The covariance matrices $Q_{k}$ and $R_{k}$ are defined as $E\left(w_{k}w_{j}^{T}\right)\;=\;Q_{k}\delta_{k-j}$ and $E\left(v_{k}v_{j}^{T}\right)\;=R_{k}\delta_{k-j}\;$, where $\delta_{k-j}$ is the Kronecker delta function; that is, $\delta_{k-j}=1$ if k=j , and $\delta_{k-j}=0$ if k≠j . The operator E(·) calculates the expectation value of a random variable [8]. The notation $w_{k}\sim N\left(0,Q_{k}\right)$ indicates that $w_{k}$ is a Gaussian randon variable with a mean of 0 and a variance of $Q_{k}=\mathrm{spur}\left(\sigma_{1..n_{x}}^{2}\right)\;$, with the standard deviation σ .

The following Equation (3) describes the discrete-time integration rule, like a Runge-Kutta or Euler integration method, and therefore must be integrated into the framework. 

(3) $$f_{k|k-1}\;=x_{k-1}+\;\int_{t_{k-1}}^{t_{k}}f\left(x_{k-1},u_{k-1}\right)\;dt.$$

### 2.2. The Extended Kalman Filter Calculation Steps

For nonlinear model state estimation the widely used EKF (extended Kalman filter) algorithm is given as a pseudo code in Algorithm 1.


**Algorithm 1**
**. EKF Algorithm with FMU evaluations**
Set k=1$\left(k\in {\mathbb{N}}^{+}\right)$$\;\mathrm{and}\;{\hat{x}}_{0}^{+}=E\left(x_{0}\right)$ $\mathrm{Set}\;P_{0}^{+}=E\left(\left(x_{0}-{\hat{x}}_{0}^{+}\right){\left(x_{0}-{\hat{x}}_{0}^{+}\right)}^{T}\right)\;$ **while** (break==false) **do** *Predict estimation by means of the FMU:* ${\hat{x}}_{k}^{-}=$$f_{k|k-1}\left({\hat{x}}_{k-1}^{+},u_{k-1}\right)\;$ $F_{k-1}=\exp\left(\underset{J_{k-1}}{\underbrace{{\partial f/\partial x|}_{{\hat{x}}_{k-1}^{+}}}}\cdot T_{s}\right)\;$ $P_{k}^{-}=F_{k-1}P_{k-1}^{+}F_{k-1}^{T}+Q\;$  *Correct estimation with current measurements:* $H_{k}={\partial h/\partial x|}_{{\hat{x}}_{k}^{-}}\;$ $K_{k}=P_{k}^{-}H_{k}^{T}\cdot {\left(H_{k}P_{k}^{-}H_{k}^{T}+R\right)}^{-1}\;$ ${\hat{x}}_{k}^{+}={\hat{x}}_{k}^{-}+K_{k}\cdot \left(y_{k}^{m}-h\left({\hat{x}}_{k}^{-}\right)\right)\;$ $P_{k}^{+}=\left(I-K_{K}\cdot H_{k}\right)\cdot P_{k}^{-}\;$ k=k+1 **end while**

The red marked sections—this holds for the following sections—indicate where the evaluation of the underlying system model equations, compare Equation (2), are necessary. The calculation of ${\hat{x}}_{k}^{-}$ is performed by integrating the prediction model Equation (1) from $t_{k-1}\;\;$to $t_{k}\;$. $F_{k-1}$ is the state-transitions matrix of f with respect to x at ${\hat{x}}_{k-1}^{+}$ and $H_{k}$ is the partial derivative matrix of h with respect to x at ${\hat{x}}_{k}^{-}\;$. The additive Gaussian noise assumption in Equation (2) is handled by the user defined covariance matrices  Q,R (the index k is removed since it is here time-invariant). The Jacobians $J_{k-1}$ and $H_{k}$ must either be provided directly (according to the FMI 2.0 specification [5], this feature is optional), or they can be determined numerically, for example with a forward difference quotient as shown in pseudo-coode in Algorithm 2:


**Algorithm 2. Numerical differential quotient determination**
1. $\mathrm{Set}\;\Delta ≅\sqrt{\epsilon }\;$ **for**
$i=1\;\mathrm{to}\;n_{x}$
**do** ${\left(J_{k-1}\right)}_{:,i}=\frac{f\left({\hat{x}}_{k-1}+\Delta \;e_{i},u_{k-1}\right)-f\left({\hat{x}}_{k-1},u_{k-1}\right)}{\Delta }\;$ **end for**

wherein ϵ is the machine precision of the particular computer architecture, ${\left(J\right)}_{:,i}$ the i-th column of J and $n_{x}$ the number of system states.

### 2.3. The Unscented Kalman Filter Sigma Point Prediction Step

The so-called sigma point transformation (SPT) is based on the idea that it is easier to approximate a Gaussian distribution than it is to approximate an arbitrary nonlinear function or transformation; see [8,29], and appendix Chapter A.4 in reference [2]. The parts of the unscented Kalman filter (UKF) algorithm, in which model evaluations are necessary, are given in Algorithm 3. The selection of the sigma points in matrix X is performed via a static scaling factor $\gamma \left(n_{x},\alpha ,\kappa \right)$ and the matrix square-root of the posteriori covariance matrix. The parameter α is the spread around the last state value ${\hat{x}}_{k-1}^{+}$ and κ is the stochastic distribution assumption. In total $2n_{x}+1$ points must be created and then used as initial values for $2n_{x}+1$ simulations from $t_{k-1}$ to $t_{k}$ to compute $X_{k|k-1}\;$.


**Algorithm 3**
**. UKF prediction step with FMU evaluations**
**1.** $\;X_{k-1}=\left[{\hat{x}}_{k-1},{\hat{X}}_{k-1}+\gamma \sqrt{P_{k-1}^{+}},{\hat{X}}_{k-1}-\gamma \;\sqrt{P_{k-1}^{+}}\right]\;$ **for**$i=1\;\mathrm{to}\;2\cdot n_{x}+1$**do** ${\left(X_{k|k-1}\right)}_{:,i}=f_{k|k-1}\left({\left(X_{k-1}\right)}_{:,i},u_{k-1}\right)\;$ **end for** $${\hat{x}}_{k}^{-}=\;\overset{2\cdot n}{\underset{i=0}{\sum }}w_{i}^{m}\cdot X_{\;k|k-1,i}$$$$P_{k}^{-}=\overset{2\cdot n}{\underset{i=0}{\sum }}w_{i}^{c}\cdot \left(X_{\;k|k-1,i}-\;{\hat{x}}_{k}^{-}\right){\left(X_{k|k-1,i}-\;{\hat{x}}_{k}^{-}\right)}^{T}+Q$$$$X_{k}^{’}=\left[{\hat{x}}_{k}^{-},{\hat{X}}_{k}^{-}+\gamma \sqrt{P_{k}^{-}},{\hat{X}}_{k}^{-}-\gamma \sqrt{P_{k}^{-}}\right]$$**for**$i=1\;\mathrm{to}\;2\cdot n_{x}+1$**do** ${\left(Y_{k}\right)}_{:,i}=h\left({\left(X_{k}^{’}\right)}_{:,i}\right)\;$ **end for**$${\hat{y}}_{k}^{-}=\overset{2\cdot n}{\underset{i=0}{\sum }}w_{i}^{m}\cdot Y_{k,i}$$

The predicted values ${\hat{x}}_{k}^{-},{\hat{y}}_{k}^{-},\;P_{k}^{-}$ are calculated via weighted sums with the predetermined weights $w_{i}^{c,m}\left(n_{x},\alpha ,\kappa \right)\;$. It is defined $\hat{X\;}:=\left[\hat{x},\hat{x},\;\ldots ,\hat{x}\right]\in$ ${\mathbb{R}}^{n_{x}\times 2n_{x}+1}$ and in the notation here a vector depending function (e.g., $f_{k|k-1}$ or h ) with a matrix argument returns a matrix with columns that are equal to the evaluated columns of the matrix argument.

It can be shown that the nonlinear approximation accuracy of the UKF is at least twice higher than the EKF. This becomes an important feature in case of strong nonlinearities in the prediction model (for a detailed proof see [9]–app. A).

In the following, a novel and automated procedure is introduced for incorporating Modelica [3] based models in nonlinear observer algorithms. The aim is to start from a given (continuous, usually nonlinear) Modelica model and automatically deduce a nonlinear observer for this model in form of a sampled data system. This task cannot be performed directly, because Modelica has no means to discretize a continuous model and to solve this discretized model with a user-defined method (that means integration and model update of the next state according to the observer equations). Note that it is insufficient to simply integrate the nonlinear models from the last to the new sample instant. Instead, the extended Kalman filter algorithm (cf. Algorithm 1) requires linearizing the model around ${\hat{x}}_{k-1}^{+}$ for the state Jacobian matrix and around ${\hat{x}}_{k}^{-}$ for the output Jacobian matrix. On the contrary, the unscented Kalman filter (cf. Algorithm 3) requires integrating the model several times with disturbed states from the last to the new sample instant. Therefore, the basic approach is to export the Modelica model in the FMI-format, import it again in Modelica and during import, call the FMI-functions in such a way that the model is discretized and utilized in a nonlinear observer algorithm.

The goal of the FMI technology is to describe input/output blocks of dynamic systems defined by differential, algebraic and discrete equations and to provide an interface to evaluate these equations as needed in different simulation environments, as well as in embedded control systems, with explicit or implicit integrators and fixed or variable step size. The FMI interface consists of a small set of standardized “C-functions” to evaluate the model equations and a XML-file that contains all information that is not needed during execution, such as the variable definitions. Every variable has a handle (a 32 bit Integer) that is used to identify the variable in the C-function calls. The source and/or object code of the C-functions, as well as the XML-file and optionally other files, are stored in a zip-file with the extension “.fmu” for “Functional Mockup Unit.”

### 2.4. Utilizing an FMU in an Estimation Algorithm

**Figure 1:**
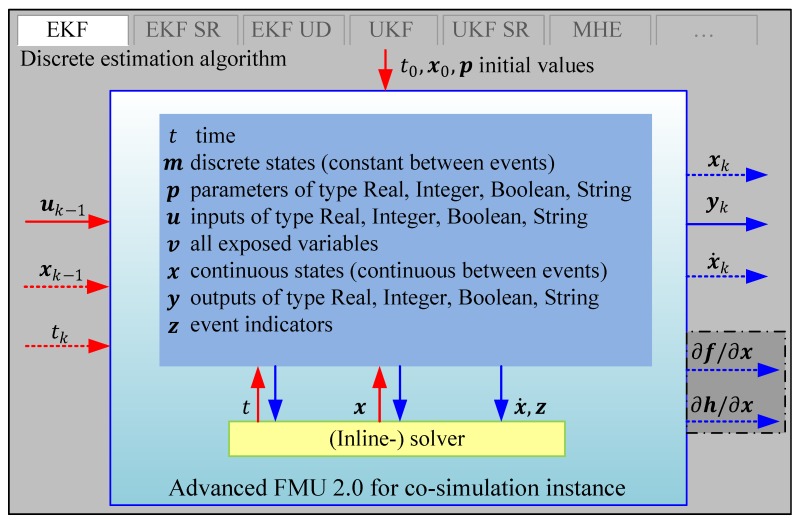
The extended FMI 2.0 for model co-simulation nested in a discrete estimation algorithm.

In Figure 1 the extended FMU 2.0 co-simulation interface embedded in the estimation framework is shown (compare Figure 10 in reference [5]). It provides the necessary interfaces for the discrete estimation algorithms to calculate the quantities described in Table 1. All non-standard FMU interfaces are indicated by dotted lines.

Besides the already explained variables t,u,x,y and the set of model parameters p (e.g., the mass of a vehicle) the FMU has the internal variables m,v,z , which are now explained briefly. The discrete states m are discrete-time variables with two values—the value of the variable from the previous event instant and the value of the variable at the actual event instant [5]. Since this work focuses on continuous-time formulated prediction models, these are not used in the prediction model FMUs. The vector v denotes all model variables, that is, states or sub-system variables. The event indicator vector z contains internal variables such as state events which give the (inline-) solver the information that an event (e.g., the fulfillment of another branch in an “if-else” statement—see for instance the vehicle’s standstill condition in [2]) occurred between the last model evaluation and the current integration step. Internally, this is handled by the (inline-) solver to guarantee a correct integration and output of the model variables. More information on this methodology can be found in Chapter 3 of [5].

**Figure 2:**
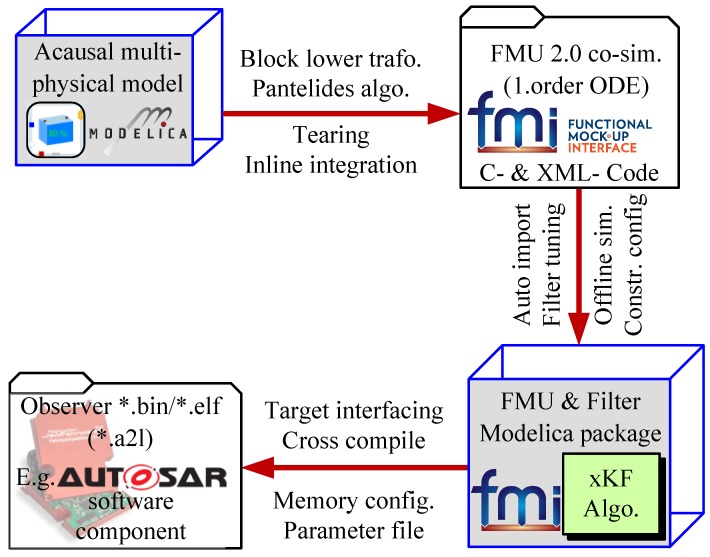
Overall model based observer generation process.

The overall process of designing a state estimator in Modelica and (cross-) compiling it for embedded targets is illustrated in Figure 2. The process can be described as follows—an acausal multiphysical Modelica model is designed in a Modelica environment such as Dymola. To fullfill requirements like real-time capability it is up to the modelling expert to find appropriate approximations, for example, for friction or hysteresis effects and to ensure it is robust simulateable with a deterministic integration algorithm such as the Runge-Kutta method. After the design process the model is translated by the simulator, for example, Dymola which performs several measures such as inlining the integration algorithm, block lower transformation and equation sorting by means of Pantelides algorithm [30,31]. Moreover, the tearing algorithm [31] is applied to reducing the size of algebraic loops to the order of one by means of substitution variables. Finally, the model is transformed in a causal first order ODE representation encapsulated in FMU 2.0 for co-simulation. This FMU is imported into the Modelica environment by extending a provided FMU interface package. For such an FMU package a set of application specific Kalman filter models is automatically generated, which makes use of the algorithmic part of the state estimator (e.g., a UKF). Finally, the individual filter model can be instantiated, parameterized or tuned in the user’s application model or can be cross compiled for an embedded target platform using the C-code sources from the FMU and a target dependent cross compiled numerical library of LAPACK (Linear Algebra PACKage, [32]), which is used for the matrix calculus operations. This process is currently under further development in the EMPHYSIS project to match the requirements (e.g., memory and word length limitations) of automotive (AUTOSAR compliant) embedded targets.

The following estimation algorithms are implemented reliably and efficiently in the DLR Kalman Filter Library—EKF (extended Kalman filter), SR-EKF (square-root EKF), UD-EKF (UD-decomposition EKF), UKF (unscented Kalman filter), SR-UKF (square-root UKF) [7,29]. Additionally, there are modified algorithms for parameter estimation as well as an extension to nonlinear moving horizon estimation (MHE) using a fast nonlinear gradient descent search as it is presented in [26]. For details on the implementation of the DLR Kalman Filter Library in the Modelica language please refer to the appendix Chapter B in reference [2].

### 2.5. Notes on Detectability and Observability

The designed prediction model is normally nonlinear and in this work there is no deeper discussion of the determination of the observability of the system. In practical applications it is mostly sufficient if the model is detectable rather than completely observable, that is, all unobservable modes/states are stable. An analysis of these properties can be performed with the DLR Modelica LinearSystems2 library [3]. As a good rule of thumb it is helpful to make a detectability and observability analysis at the nominal operation points or in a grid of potential operation modes.

## 3. Kalman Filter Theory Extension with Inequality Constraints

The so far presented estimation algorithms, based on the Kalman approach, are very well suited for a large bandwidth of observer problems. Besides the stochastic assumption of zero mean, Gaussian white noise processes, there are neither system state constraints nor delays in the data acquisition chain considered. In [33] a good and comprehensive overview of existing methods is given for incorporating (linear) constraints to Kalman filtering for linear and nonlinear systems. Some of these methods are not only applicable for linear constraints and they can also handle nonlinear constraints. Especially the estimation projection methods can be used for nonlinear constraints through a first or second order Taylor series expansion around the a priori state estimation ${\hat{x}}_{k}^{-}\;$. For the approach of perfect measurements (PM) constraint handling, this linearization is repeatedly applied to the measurements to smoothly improve the estimation.

This article focuses on the class of nonlinear inequality constraints for nonlinear state estimation, since for many estimation tasks the control design engineer needs to incorporate inequality constraints to the states, for example, the slider position state s of an inverted pendulum is limited to the interval $s\in \left[s_{\min},s_{\max}\right]\;$. Moreover, the constraints should be formulated (non-)linear by means of the Modelica language within the prediction model or in a separate FMU. Additionally, by reason of exchangeability and flexibility, the state constraint handling algorithms should be separable from the particular unconstrained Kalman filter algorithm.

That is, the user can configure the estimation task with constraint handling features and then examine different Kalman filters (e.g. EKF or UKF) without the need to adopt the constraints to the particular estimation algorithm. For these reasons it has been decided to extend the posteriori estimation projection approach with an inequality constraints algorithm in a computationally efficient way to guarantee real-time capability. This is explained in the following subchapters.

To the best knowledge of the author a new method for nonlinear estimation with nonlinear inequality constraints is given in the next chapter (i.e., computational simplified nonlinear inequality constraints handling for the case that one constraint is active) that is different to the algorithms in literature (see Table 2). The following proposed estimation projection method is appropriate for nonlinear inequality constraints of the form c(x)≤0 , with limits to the distinguished approximation. First, in Chapter 3.1 an efficient root-finding algorithm determines a linear scaling factor between the vector distance $\bar{{\hat{x}}_{k}^{+}\;{\hat{x}}_{k-1}^{+}}$ to the point where the constraint gets active c(x)=0 . Afterwards, a different method is proposed, with a Lagrange multiplier. This method determines a constrained estimate that lays closer to the border of the constrained surface c(x)=0 .

### 3.1. The Formulation of State Constraints c(x)≤0

In the most common cases there exist a lot of known constraints to estimated states, for example, a limitation of the position due to physical stops in a mechanical system. These can be formulated as restrictions that fulfill a set of inequalities c(x)≤0 . The main principal of the proposed algorithms is to change the state vector after the correction step ${\hat{x}}_{k}^{+}$ of the Kalman filter such that all restrictions are fulfilled. The optimization problem is defined as:

(4) $$\begin{array}{c} {\hat{x}}_{k}^{0}=\underset{x}{\mathrm{argmin}}\|x-{\hat{x}}_{k}^{+}\|\\ \;s.t.c\left(x\right)\leq 0 \end{array}$$

It is assumed that the constraints c(x)≤0 are neither contradictory nor redundant and only one constraint is active at the same time. In many cases this is valid, since the estimation step size is small and, for example, the constraints are designed as bands without complex overlap. Examples for this are road boundaries in a position estimation problem cf. [2]. In case of large step sizes and/or if multiple constraints are likely to be active, it is possible to handle these situations by an sequential quadratic program (SQP) formulation (see Table 2). An SQP approach can be seen as more general but also computationally more complex in comparison to the here proposed problem-specific approaches. This is justified by the necessity in the SQP algorithm of an iterative search to determine the Lagrange multiplier by solving QP sub-problems [34]. Moreover, the Hessian of the QP needs to be approximated, also in an iteratively manner, by means of a Broyden–Fletcher–Goldfarb–Shanno algorithm BFGS approach [37]. The BFGS algorithm is a quasi-Newton method which approximates the Hessian matrix using updates specified by evaluations of the gradient.

**Figure 3:**
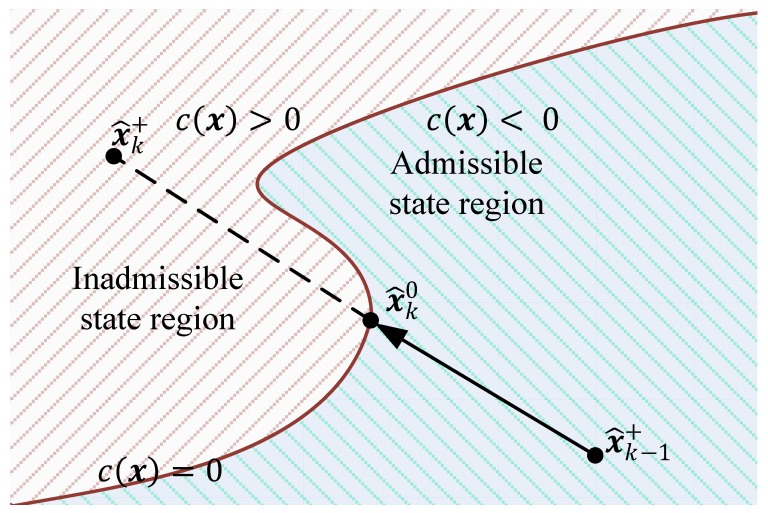
Barrier violation in constrained state estimation.

The obvious way to handle the active constraint is to find the point ${\hat{x}}_{k}^{0}$ in the vector $\bar{{\hat{x}}_{k}^{+}\;{\hat{x}}_{k-1}^{+}}$ where the violated constraint c(x)>0 is no longer fulfilled (see Figure 3). This means the root α of c(x(α))=0 has to be determined, such that

(5) $${\hat{x}}_{k}^{0}={\hat{x}}_{k-1}^{+}+\alpha \cdot \left({\hat{x}}_{k}^{+}-{\hat{x}}_{k-1}^{+}\right)\;,\alpha \in \left[0,1\right]$$

This problem is solved with the derivative free root finding method of Brent [38]. This algorithm is available in the Modelica Standard Library (“Modelica.Math.Nonlinear.solveOneNonlinearEquation”) [3]. Once α is known, the Cholesky decomposition $CP_{k}^{0}$ of the covariance matrix can be calculated as:

(6) $$CP_{k}^{0}=\left(1-\alpha \right)\cdot CP_{k}^{-}+\alpha \cdot CP_{k}^{+}$$

The rudimentary way would be to use this solution for the estimation projection, but one can easily see in Figure 3 that it is not the closest estimate to the unconstrained solution ${\hat{x}}_{k}^{+}\;$. Therefore, two approaches are discussed in the following that are intended to find a better approximation of the solution.

### 3.2. The Simplified Newton Descent Search Approach

As a second approach, a more general method for incorporating inequality constraints is presented. Different to this a sigma point projection based algorithm (cf. Table 2), as proposed in [2] and [36], works fine with the SR-UKF as well as the SR-EKF algorithm, because in these cases the algorithm can directly use the Cholesky factorization of the a priori covariance matrix. For other algorithms that do not rely on square-root filtering [29], a computational costly Cholesky decomposition of the covariance matrix would have to be computed in every time instant when a constraint gets active. The here proposed algorithm is based on a simplified Newton descent search (i.e., without considering the second order derivative), that projects the posteriori estimation on the constrained surface. This opens up a more general use. The optimization objective is formulated as follows:

(7) $$\begin{array}{c} {\hat{x}}_{k}^{P}=\;\underset{x}{\mathrm{argmin}}\|x-{\hat{x}}_{k}^{+}\|\\ s.t.c\left(x\right)=0 \end{array}$$

**Figure 4:**
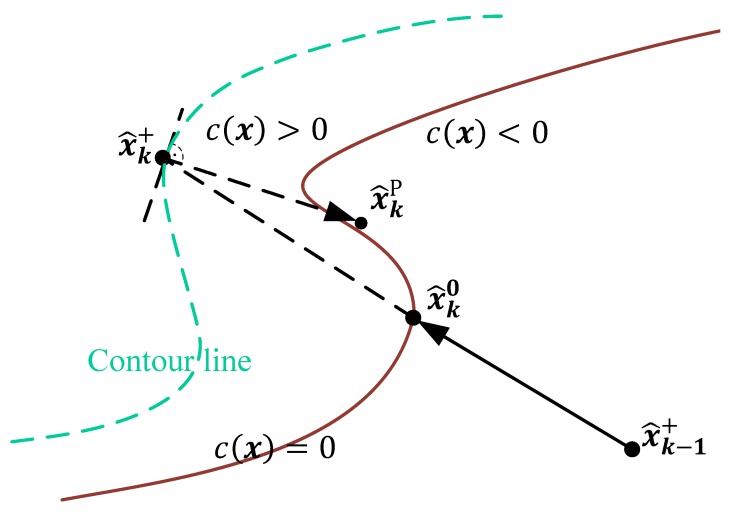
Principle of the Newton descent search for state constraint handling.

The idea behind this algorithm is to perform a descent search along the gradient $\nabla c\left({\hat{x}}_{k}^{+}\right)$ calculated at the point ${\hat{x}}_{k}^{+}$ until the constraint equation $c\left({\hat{x}}_{k}^{P}\right)\leq 0$ holds again. Graphically this can be interpreted as shown in Figure 4.

This optimization task can be formulated with the method of Lagrange multipliers. Equation (8) denotes the above formulated optimization objective in a general description:

(8) minℒ(x,μ)=f(x)−μ⋅(g(x)−d)

Therefore, the optimal solution of this unrestricted minimization problem is denoted as follows:

(9) $$\nabla_{x,\mu }ℒ\left(x,\mu \right)=0$$

This approach is now applied to the estimation projection optimization problem Equation (7) and the searched restricted estimate ${\hat{x}}_{k}^{P}$ is calculated using the Lagrange multiplier μ and the gradient of the active constraint c(x) :

(10) $$\begin{array}{c} {\hat{x}}_{k}^{P}={\hat{x}}_{k}^{+}+\mu \cdot \nabla c\left(x\right)\;s.t.c\left(x\right)=0\\ \;c\left({\hat{x}}_{k}^{+}+\mu \cdot \nabla c\left(x\right)\right)=0 \end{array}$$

To fulfill Equation (10) the approach is formulated with a simplified Newton descent search algorithm by means of a scalar zero search F(μ)=0 with respect to the Lagrange variable μ :

(11) $$\begin{array}{c} \nabla c\left(x\right)\approx \nabla c\left({\hat{x}}_{k}^{+}\right)\\ \;\overset{\mathrm{yields}}{\to }F\left(\mu \right)=c\left({\hat{x}}_{k}^{+}+\mu \cdot \nabla c\left({\hat{x}}_{k}^{+}\right)\right)\overset{!}{=}0\\ \;\;\frac{\partial F}{\partial \mu }=\nabla c{\left({\hat{x}}_{k}^{+}+\mu \cdot \nabla c\right)}^{T}\cdot \nabla c\left({\hat{x}}_{k}^{+}\right)\\ {\frac{\partial F}{\partial \mu }|}_{\mu =0}=\nabla c{\left({\hat{x}}_{k}^{+}\right)}^{T}\nabla c\left({\hat{x}}_{k}^{+}\right) \end{array}$$

This can be implemented as an iterative search algorithm, which determines μ such that the condition F(μ)=0 holds within a predefined maximum number of calculation steps. The pseudo code for the algorithm is given in Algorithm 4.

**Algorithm 4**. Simplified Newton descent search algorithm for constrained estimation projection.**1.** $\mathrm{Set}\;\mu_{o}=0$ and k= −1 and iter=1 and max_iter=30 $\mathrm{Set}\;\alpha ={\left(\nabla c{\left({\hat{x}}_{k}^{+}\right)}^{T}\cdot \nabla c\left({\hat{x}}_{k}^{+}\right)\right)}^{-1}\;$ **while**$\left|\alpha \cdot c\left({\hat{x}}_{k}^{+}+\mu_{k+1}\cdot \nabla c\left({\hat{x}}_{k}^{+}\right)\right)\right|\geq \epsilon_{\mathrm{Newton}}$ and iter < max_iter
**do**  k=k+1   $\Delta \mu_{k+1}=-\alpha \cdot c\left({\hat{x}}_{k}^{+}+\mu_{k}\cdot \nabla c\left({\hat{x}}_{k}^{+}\right)\right)\;$  $\mu_{k+1}=\;\mu_{k}+\Delta \mu_{k+1}\;$  iter=iter+1 **end while** *Solution:*
$${\hat{x}}_{k}^{P}={\hat{x}}_{k}^{+}+\mu_{k+1}\cdot \nabla c\left({\hat{x}}_{k}^{+}\right)$$


## 4. A Constrained Nonlinear Battery Observer

This section is an extended version of [13] and [28], which addresses the modeling of a lithium-ion cell for online monitoring and offline benchmarking purposes. It is based on a modified version of the enhanced self-correcting model (ESC) originally proposed by [24], which is an equivalent circuit model with one RC element in parallel and an additional hysteresis voltage source (compare Figure 2c in reference [39]). This physical lithium-ion cell model is combined with smoothly interpolated lookup table of cell characteristic information from laboratory tests. The model is fully parameterized and validated with the cell type used in the high voltage (HV) battery pack of the robotic battery electric vehicle DLR ROboMObil [1]. It benefits from automatic discretization with higher order solvers, lookup table interpolation, as well as derivative calculation and symbolic transformation by means of the Modelica simulator (cf. Chapter 2), as well as from the derivative-free unscented Kalman filter (Chapter 2.3) with inequality state constraints (Chapter 3).

### 4.1. An Equations and Grid Table Combined Cell Model

In the following section, a modified and enhanced version of the ESC model is developed, referred to as the modified enhanced self-correcting model (MESC). In contrast to the original approach the prediction model of the single lithium-ion cell is formulated in acausal continuous-time representation using Modelica. This mathematical description benefits from the automated discretization of the model, by means of a Modelica compiler. The generated prediction FMU can incorporate higher order real-time capable integrators, for example, a Runge-Kutta 4 method and therefore the accuracy as well as stability for a larger sample time $T_{s}\;\;$can be significantly improved. The mathematical description of the MESC model in nonlinear state-space representation is formulated as follows:

(12) $$\left[\begin{array}{c} \dot{l}\\ \dot{h}\\ \dot{f_{f}} \end{array}\right]=\left[\begin{array}{c} -\frac{\eta_{\mathrm{Ah}}\cdot k_{i}}{C_{N}}\cdot i_{\mathrm{cell}}\\ \left|\frac{\gamma \cdot \eta_{\mathrm{Ah}}\cdot k_{i}}{C_{N}}\cdot i_{\mathrm{cell}}\right|\cdot \left(M-h\right)\\ -\omega \cdot f_{f1}+\omega \cdot i_{\mathrm{cell}}\\ \omega \cdot f_{f1}-\omega \cdot f_{f2}\\ \omega \cdot f_{f2}-\omega \cdot f_{f3}\\ \omega \cdot f_{f3}-\omega \cdot f_{f4} \end{array}\right]$$

**Figure 5:**
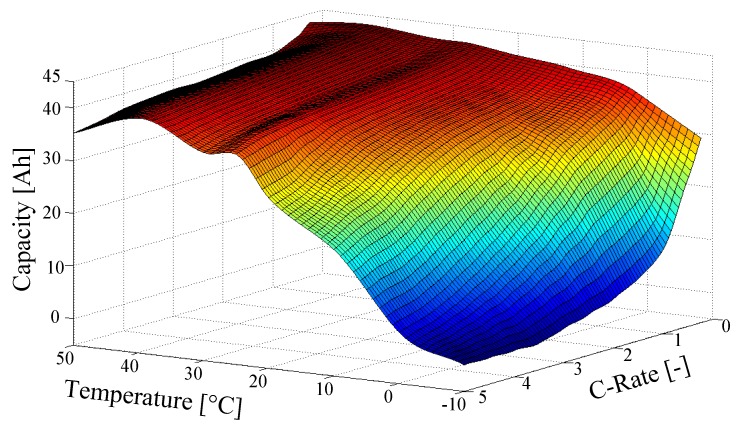
ROboMObil’s measured cell capacity in dependency of temp. and current *C_Rate_ = C_N_/l.*

The differential equation for the SOC l depends on the cell current $i_{\mathrm{cell}}\;$, the nominal cell capacity $C_{N}\;$, the coulombic efficiency $\eta_{\mathrm{Ah}}$ and a correction factor $k_{i}\;$. This factor takes the variation of the cell capacity into account due to the (dis)charging rate and the temperature, see Figure 5 The C-rate is a normalized measure of electric current, defined as the ratio of current *I(t)* in Amperes, to a cell’s nominal capacity $C_{N}$ in Ampere-hours [18]. Following [40] the correction factor is determined by:

(13) $$k_{i}=\{\begin{array}{c} c_{i}\cdot i_{\mathrm{cell}}+k_{0},\;\;\forall \;i_{\mathrm{cell}}>0\;\left(\mathrm{chrg}.\right)\\ e^{c_{i}\cdot i_{\mathrm{cell}}}+\left(k_{0}-1\right),\;\;\forall \;i_{\mathrm{cell}}<0\;\left(\mathrm{dischrg}.\right) \end{array}$$

wherein $c_{i}$ is a positive constant leading into a straight line for positive cell current, which intersects the ordinate at $k_{0}\;$. For a negative cell current the correction factor is described by an exponential function. The parameters $c_{i}$ and $k_{0}$ are determined from capacity tests replacing the simple straight line with more accurate look-up tables.

The hysteresis voltage h (presented in the second differential equation in (12)) is described by a more complex equation considering the additional factors M (polarization voltage) and γ (time constant). It describes the dynamic influence of charging and discharging of the cell as depicted in Figure 7. Herein M is half of the difference between the charge (blue) and discharge (red) line in dependency of the SOC, exemplified for l=0.2 . Especially for lithium-ion cell types with a very flat SOC/OCV curve characteristic (e.g., LiFePO_4_ cell chemistry) neglecting this dynamics may lead to a major model error. The remaining four differential equations describe an optimized fourth order critical damping current filter with only one remaining parameter ω and its states $f_{f}\;$

(14) $$u_{\mathrm{cell}}=U_{\mathrm{OCV}}\left(l\right)+h-R_{i}\cdot f_{f4}$$

**Figure 6:**
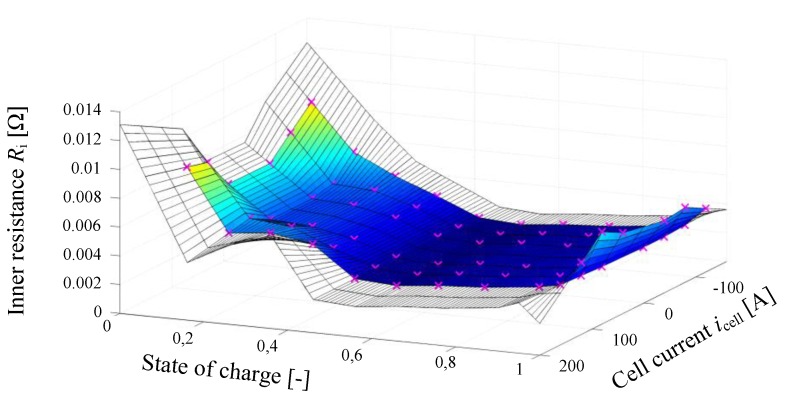
Grid table of internal cell resistance for *T = 25 °C.*

**Figure 7:**
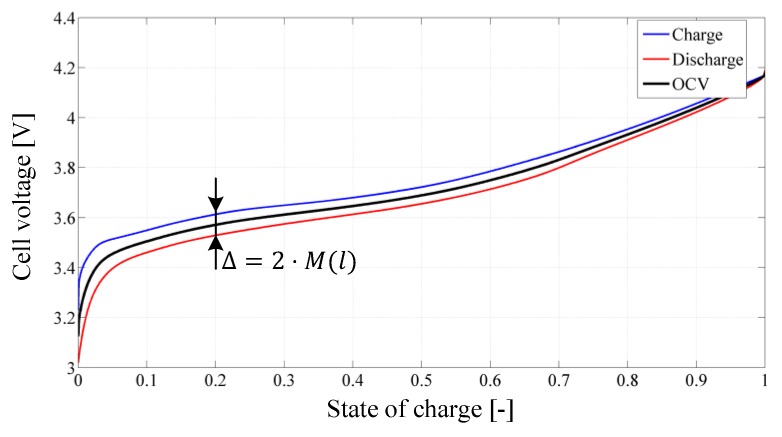
Characteristic of the cell open circuit volt. and hysteresis at *T = −10 °C.*

The output Equation (14) is similar to the original ESC model’s output equation but aggregates the influence of the cell current (ohmic loss and current filter) into one summand. The internal resistance $R_{i}\left(l,i_{\mathrm{cell}},T\right)$ of the cell is one of the most important descriptive variables. It depends on the SOC, the cell current and the temperature resulting in a three dimensional look-up table. This relationship is visualized for room temperature (T=25 °C ) in Figure 6. Considering Equation (14) it is obvious that the resulting cell voltage $u_{\mathrm{cell}}$ varies highly in case of a low SOC and high current flow due to an internal resistance increase of the cell. In such cases the cell is in a demanding situation and can be damaged irreversibly, since the higher resistance causes intense internal cell heating. Another important cell variable is the open circuit voltage (OCV) $U_{\mathrm{OCV}}\;$, whose characteristic curve incorporates the relationship between SOC and OCV, as shown in Figure 7. This figure also illustrates the hysteresis effects during charging and discharging of the cell. The blue and red curve are measured in a cell test bench experiment with very low currents applied to the cell terminals.

This minimizes excitation of the cell dynamics so that the cell terminal voltage can be considered unloaded. In addition, the influence of the internal resistance is eliminated during the data analysis. The polarization voltage M is defined as half of the difference between the two curves and therefore also depends on the OCV. 

**Figure 8:**
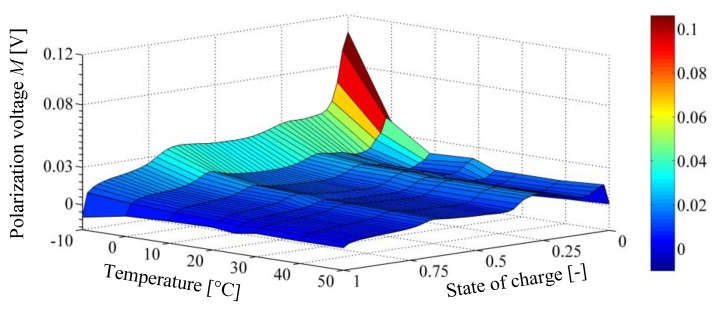
Cell polarization voltage M in dependency of temperature and state of charge.

Tests have shown that the polarization voltage depends on the actual cell temperature, whereas the highest effect of the considered lithium-ion cell type is for temperatures below 0 °C . However, it is important to mention that the hysteresis effect at positive temperature might be more significant for other cell types (e.g., ${\mathrm{LiFePO}}_{4}$ or NiCoAl). This relation is presented graphically in Figure 8.

### 4.2. Cell Model Parameter Derivation and Validation

**Figure 9:**
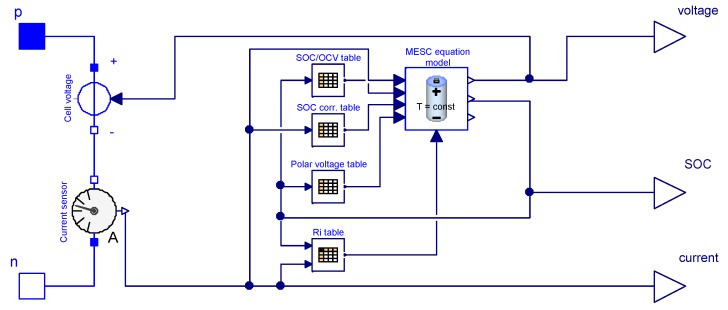
Modelica model of the MESC based on grid tables and equations.

In the context of the ROboMObil project, it was possible to obtain a high performance cell from Li-Tec Battery GmbH “Li-Tec HEI40”. It has a nominal capacity of 40 Ah and with its safety features it is fully capable for series production. For details on technical specifications please refer to Appendix A. All cell measurements for parameterization, testing, and validations were done with a Vötsch “VT4011” environment simulator and a BaSyTec cell testing system. In Figure 9 the Modelica implementation of the MESC model is shown. The stationary grid table data SOC/OCV, SOC correction, Polar Voltage, and $R_{i}\;\;$have been derived offline with this experiment setup.

The free model parameters in Equation (12), by name γ (hysteresis voltage change rate) and $f_{f_{1..4}}$ (input current filter parameters) were determined by optimization with DLR MOPS (see [41] for more information) on the Linux cluster of the DLR SR institute.

### 4.3. Kalman Filter Setup – Perfect Measurements

The MESC model has one input, the cell current, and one output, the cell voltage. These two quantities can be measured directly with high accuracy even in embedded systems. The third quantity is the cell temperature. It is determined by the use of a thermocouple sensor on the cell surface. To achieve a better estimation performance, a second measurement equation is implemented. The main idea is to take constraints into account with a recursive Kalman filter. For this purpose, an additional fictitious measurement is introduced. It can be weighted through the tuning of the output covariance matrix of the Kalman filter. This method is known as perfect measurement (PM) in the literature [33]. In this way the output equation of the MESC model is extended to:

(15) $$y=\left[\begin{array}{c} u_{\mathrm{cell}}\\ l \end{array}\right]$$

The first equation is identical to Equation (14) and the second one can be derived as follows:

(16) $$\begin{array}{c} u_{\mathrm{cell}}\approx U_{\mathrm{OCV}}\left(l\right)-R_{i}\cdot i_{\mathrm{cell}}\\ \;\Rightarrow U_{\mathrm{OCV}}\left(l\right)\approx u_{\mathrm{cell}}+R_{i}\cdot i_{\mathrm{cell}}\\ \Rightarrow \;l\approx l_{\mathrm{meas}}=U_{\mathrm{OCV}}^{-1}\left(u_{\mathrm{cell}}+R_{i}\cdot i_{\mathrm{cell}}\right) \end{array}$$

The measured SOC is calculated through the inverse of the OCV $U_{\mathrm{OCV}}^{-1}$ lookup table in combination with a low pass filter to prevent peaks in the perfect measurement output due to rapid changes of the input current. This extension allows the Kalman filter to adjust the SOC directly and therefore to enforce a physically correct estimation.

### 4.4. Observer Configuration on an Embedded Microcontroller 

**Figure 10:**
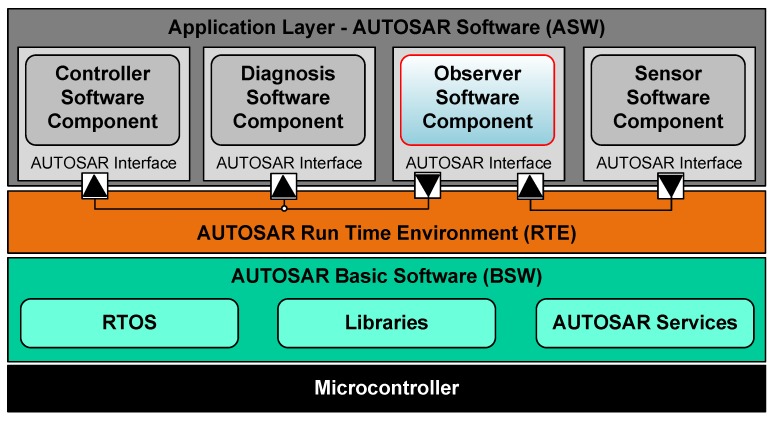
Schematic of an AUTOSAR layered architecture with an observer software component.

To exemplify the usage of a cell battery observer on an embedded microcontroller an AUTOSAR architecture has been chosen since it is subject of current research at DLR SR within the ITEA EMPHYSIS project and it is getting more and more important in the field of automotive. In the style of [42] a schematic configuration of an AUTOSAR layered microcontroller configuration is sketched in Figure 10. On the lowest layer, the microcontroller interacts with it’s I/Os and other controllers via a bus system, for example, via CAN, or receives electrical signals from sensors or gives electric commands to actuators, for example, via the power electronics of an electric machine. In the overlaying turquoise colored layer the AUTOSAR Basic Software layer is arranged. In this layer bit streams from the I/O are more and more abstracted by help of different modules and a real-time operation system (RTOS) in combination with various libraries. These represent an infrastructure for the execution of components on the top layer, the AUTOSAR Software application layer. Between these both lies a middle layer, the AUTOSAR run time environment, which provides all interfaces and in-/outputs, between the lower hardware abstraction and the top application layer. The exchange between these both is described by a *.arxml file. Details on this are given, for example, in [42]. By this separation of application and close to hardware programming it is possible to design novel model and FMI based algorithms without the need to have deep knowledge of the complex AUTOSAR architecture. In the application layer of Figure 10 an example configuration with different applications, which make use of the here-proposed battery observer, is shown. In this example, a sensor software component performs signal preconditioning such as scaling, faulty sensor detection and initialization. Then, by means of an FMI based observer algorithm the observer software component (subject of investigation in this project) calculates the complete set of (virtual) sensor signals that then can be used within a controller or diagnosis software component.

This observer software component should embed the extended Kalman filter algorithm with nested FMUs for the cell model (MESC) and perfect measurements (PM), that is, in this scenario the FMUs are not interconnect-able to other software components as it is proposed in [42] and are only available to the Kalman filter algorithm.

**Figure 11:**
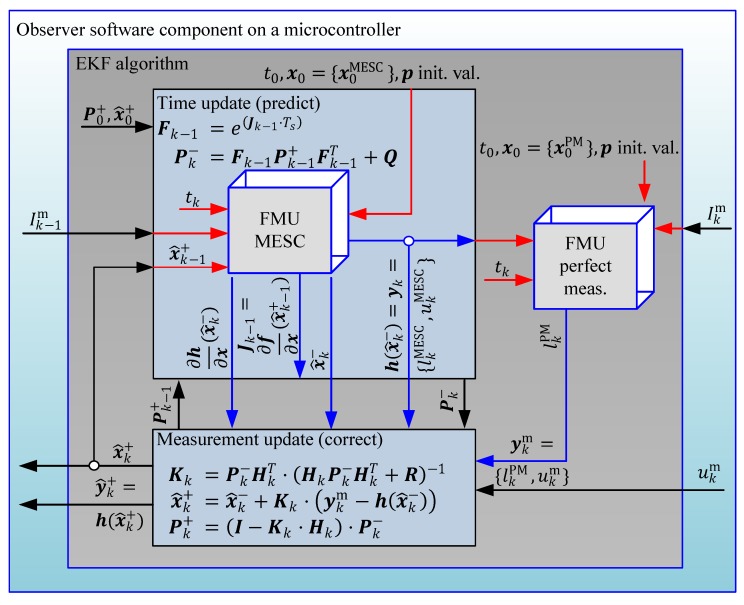
Schematic of the cell observer software component and it’s interaction with the FMUs.

In Figure 11 the battery observer control structure is schematically pictured with the signal flow between the components and the FMUs as well as the algorithmic evaluations in the Kalman filter steps according to Figure 1 and Algorithm 1. The inputs, that need to be provided by the RTE, are the measured battery current $I_{k-1}^{m}$ and cell terminal voltage $u_{k}^{m}\;$. As outputs to the RTE the complete estimated state vector ${\hat{x}}_{k}^{+}$ (compare Equation (12)) and the corrected output vector ${\hat{y}}_{k}^{+}$ (compare Equation (15)) are defined. After the initialization phase with user tune-able model and filter parameters of the EKF as well as the both FMUs (MESC and perfect measurements), all indicated with the lower index ${\left(\cdot \right)}_{0}\;$, the algorithm is cyclic executed with a predefined fixed cycle time $T_{s}=t_{k+1}-t_{k}\;$. In the prediction step a forward integration with the previous state estimates ${\hat{x}}_{k-1}$ and the input $I_{k-1}^{m}$ is calculated by the FMU MESC. Moreover, the directional derivatives of the states and the FMU outputs are computed, to determine the covariance prediction $P_{k}^{-}$ of the Kalman filter. In the second step the perfect measurement FMU is evaluated to calculate a value for the fictive measured SOC $l_{k}^{\mathrm{PM}}$ (Equation (16)) that can be incorporated in the correction step of the Kalman filter. The inputs to the FMU PM are { $l_{k}^{\mathrm{MESC}},I_{k}^{m},u_{k}^{\mathrm{MESC}}\}\;$. It is also valid to use $u_{k}^{m}$ instead of $u_{k}^{\mathrm{MESC}}\;$, but the predicted cell voltage is less noisy and therefore preferable to the sensor signal. In the third and final step the measurement update of the Kalman filter is computed. The measurement vector $y_{k}^{m}$ is composed of the fictive SOC measurement $l_{k}^{\mathrm{PM}}$ and the actual measured cell terminal voltage $u_{k}^{m}\;$, the covariance matrix $P_{k}^{+}$ is updated and the corrected state vector ${\hat{x}}_{k}^{+}$ is computed. The latter two values are used in the next cycle $t_{k+1}$ of the time update step as recursion measures.

### 4.5. Parameterization and Model Validation

**Figure 12:**
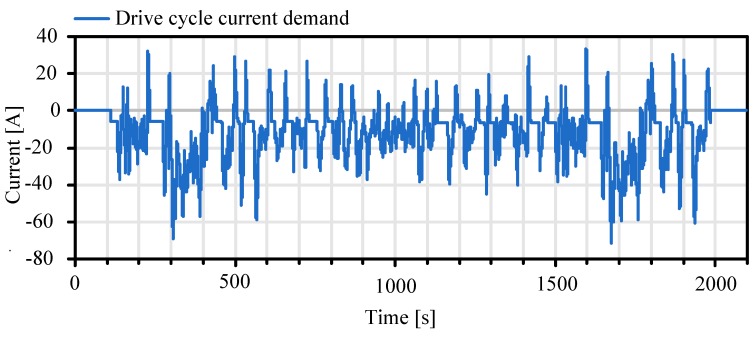
Calculated cell current demand based on FTP-75 drive cycle.

In this chapter the experimental results with the unconstrained battery observer are discussed. For the generation of the experiment data a FTP-75 driving cycle is used as driving demand for ROboMObil’s longitudinal dynamics powertrain model which is based on the DLR PowerTrain Library [3]. This model and its resulting power demand calculation have been validated on the DLR roller test rig in Stuttgart with the whole battery pack (cf. [1]). With this multiphysical model it is possible to convert the calculated electric power demand of the actuators into the current demand of one cell. In a next step this current demand (Figure 12) is used as stimuli data for a single cell test bench in a climate chamber.

The voltage at the cell terminals, the surface temperature and the effective current flow are recorded during this test. Finally, they are used as input and measurement data for the nonlinear battery observer experiment setup, shown in Figure 11.

**Figure 13:**
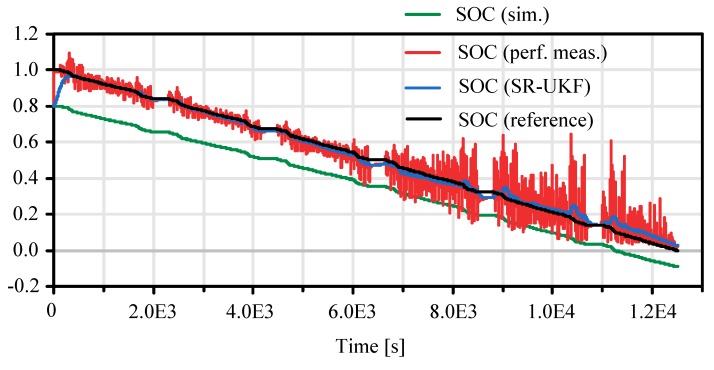
Unconstrained observer based state of charge estimation.

In Figure 13 the experimental and the corrected results of the proposed FMI model based real-time observer are presented. The black reference curve is determined via precise testbench experiment with high fidelity current measurements. The red curve shows the SOC characteristic calculated via the perfect measurement. 

It is very erratic and noisy, in spite of signal prefiltering. This characteristic is qualitatively correct, especially in comparison to the green curve, which represents the output of a pure model simulation without observer correction. The pure simulation yields a SOC that is less than zero at the end of the simulation which is physically impossible (cf. Figure 13, bottom right). In a real world automotive application, this would cause the SOC display to show incorrect information. In this case it would not be possible to drive on, although the battery is not yet exhausted. Through the nonlinear estimation algorithm, a better and smoother estimation of the SOC can be achieved that converges to zero at the end (blue curve). These observations are quantified by normalized root mean square error criteria (FIT) in the following table. The results of the SOC SR-UKF with an average deviation of ~2–3% SOC to the reference true value grant a good result of this estimator compared to recent developments in state of charge estimation as Figure 8 in reference [43] for LiNMC cell types (same chemistry as used here—see Appendix A). Further optimization of the Kalman filter parameters and reducing the sensor noise may also improve the results.

Due to the efficient code for the prediction model provided by the extended FMI 2.0 co-simulation interface, this estimator runs with a real-time factor greater than 100 on standard desktop systems (Intel i7-4600U 2,7 GHz, 8 GB Ram, SSD). Thereby, it was possible to implement the observer on an embedded rapid prototyping controller of ROboMObil.

### 4.6. Extension to Inequality Constraint SOC Estimation

In this section the application of the proposed constraint handling mechanisms (see Chapter 3) for an one-step Kalman filter estimation algorithm is exemplified. Both approaches, a sigma point projection approach (cf. [2]) as well as the simplified Newton descent search (Chapter 3.2), can be used here since a SR-UKF filter has been selected as the most appropriate method (cf. Table 3), which is stable in case of this estimation task. A common problem in battery state of charge estimation is the limitation of the SOC to realistic values at least between zero and one. Misinterpreted values of the perfect measurement Equation (16) exceed the abovementioned boundary values and may cause wrong values of the available charge and therefore also of the power allocation budget. A simplified approach to handle this is to limit the output value of the estimation, but this causes wrong values for future SOC estimate since the state tends more and more in the inadmissible region.

**Figure 14:**
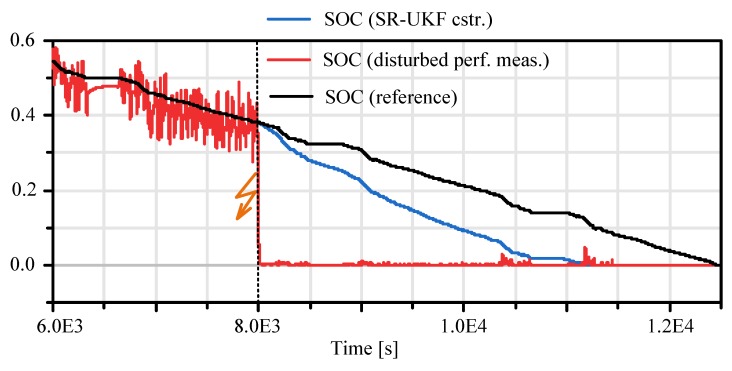
Constrained observer based state of charge estimation.

**Figure 15:**
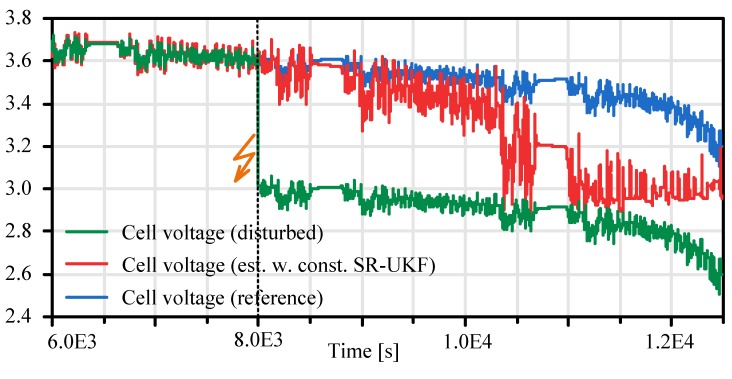
Constrained observer based cell voltage.

In Figure 14 the influence of the proposed constraining methods is shown. In this scenario a sensor failure is virtually generated that causes a static cell voltage measurement drop of ΔV=0.6V at $T=5\cdot 10^{3}\;s$ (cf. Figure 15). Due to this the fictive SOC measurement calculated by the PM (cf. Chapter 4.3) drops immediately to zero (cf. Figure 14–$T=8\cdot 10^{3}s\;$). The estimated SOC follows this trend more smoothly until it reaches also a SOC of zero at $T=1.1\times 10^{3}s\;$, where it is limited by the constraining algorithm. In Figure 15 (red curve) it can be seen that not only the SOC is effected by constraining algorithm also the observer output cell voltage is limited to the end-of-discharge voltage of the cell. 

This simple example should give a motivation for the usage of the here proposed algorithms, in combination with a sensor fault detection system (cf. Figure 10) this methodology can help to prevent suddenly occurring faulty system states (i.e. immediately SOC drop to zero cf. Figure 14).

Both state constraining methods have shown similar results in their temporal characteristics (see Table 4), the main difference could be found in the mean integration time ${\bar{t}}_{k}\;$, where the sigma point projection methods outperforms the more general simplified Newton descent search approach. The experiment with a simulation period of $1.2\cdot 10^{4}\;s$ has been executed on a 64-bit Windows based system (Intel i7-4600U 2,7 GHz, 8 GB Ram, SSD).

## 5. Conclusions

This work presented a new highly automated framework for generating model based observers for use in embedded applications. The benefits in comparison to conventionally manual and application-specific observer implementation can be summarized as follows:

- Representation of a nonlinear acausal prediction model in an object oriented multi-physical modeling language (Modelica).
- Automated, well proven and efficient equation sorting and discretization in a standardized format;
- Direct integration in a library containing different types of nonlinear Kalman filter algorithms.
- Formulation of nonlinear state constraints in the same way as the prediction model.
- Proposal of a simplified approach for handling nonlinear state constraints.
- Automated code generation for a cross-platform embedded target.

Moreover, the observer framework along with the state constraint mechanism have been successfully applied to a battery electric vehicle state of charge estimation problem. The results and benefits of the nonlinear constrained cell observer can be concluded as follows:

- A continuous-time Modelica semi-physical cell model with state dependent characteristic mapping correlation has been automatically symbolically manipulated and discretized via Dymola and imported into the model based observer framework via the extended FMU 2.0 co-simulation interface.
- This gives benefits in comparison to [24,25] by way of error-prone manual discretization, the capability to make use of higher order discretization methods as well as prediction model integrated efficient table interpolation.
- The cell prediction model parameters and characteristics were derived from real world test bench experiments matching the cell type used in ROMO for the high voltage system.
- An SR-UKF observer extended with a computional reliable method for state constraint handling has been designed and validated with experimental data from cell test bench investigations.

## 6. Outlook

It is planned to release a commercial version of the DLR Kalman Filter Library as a Modelica Library as well as a MATLAB compliant version by the end of 2019. Ongoing research in the EMPHYSIS project will focus on an embedded platform specific version of the FMI standard, special numeric routines tailored to single precision computing and extended problem formulations such as NMPC or nonlinear dynamic inversion. Moreover, for future development it will be investigated, whether a software component design with a separation of the observer algorithm component and the underlying FMUs in different software components should be preferable (similar to [42]). 

## Figures and Tables

**Table 1:** Model evaluations by means of the FMI for nonlinear state estimation.

Model Evaluations	
Integration between two sample points:	$\;f_{k|k-1}=\;x_{k-1}+\;\overset{t_{k}}{\underset{t_{k-1}}{\int }}f\left(x,u_{k-1}\right)\;dt\;$
Derivative evaluation:	$\;\dot{x}=f\left(x,u\right)\;$
Output evaluation:	y=h(x)
State Jacobian matrix (optional):	$\;\frac{\partial f}{\partial x}\left(x,u\right)\;$
Output Jacobian matrix (optional):	$\;\frac{\partial h}{\partial x}\left(x\right)\;$

**Table 2:** Nonlinear constraint handling methods for nonlinear Kalman filters.

Method	Approach Description
Nonlinear soft constraints	Nonlinear soft constraints by means of perfect measurements [33] are shown in the application example of a battery state estimator in Chapter 4.
Nonlinear optimization based estimation projection	Project the states on the constrained surface by solving the restrictive optimization problem $\underset{x}{\min}\|x-{\hat{x}}_{k}^{+}{\|}_{W}\;\;\;\;\;s.t.\;\;d\left(x\right)=0,\;c\left(x\right)\leq 0$ it can be solved by means of a nonlinear interior point solver or a sequential quadratic program (SQP) [34].
Sigma point projection	This is a special approach for UKF algorithms. The sigma points are projected on the borders of the constrained region and with these projected points the covariance update is calculated [35]. In [36] and [2] additionally the sigma point weights are scaled to improve the constrained covariance confidence. Similar methods are the two-step UKF and the unscented recursive nonlinear dynamic data reconciliation (URNDDR) which performs a MHE with a horizon size of one to the posteriori sigma points [33].
Nonlinear moving horizon estimation	A nonlinear moving horizon estimator with a nonlinear gradient descent optimization which can handle equality and inequality constraints as well as delayed measurements is formulated [2]. Besides this, various other formulations, like the URNDDR, the constrained UKF [2] or the constrained real-time approach using the ACADO toolbox [15] can be found as examples for nonlinear moving horizon estimation.

**Table 3:** State of charge estimation assessment by means of the FIT criterion.

SOC Reference Compared to	FIT
SOC SR-UKF	90.3649%
SOC SR-EKF	88.1267%
SOC perf. measurement	83.3738%
SOC simulation	47.5859%

**Table 4:** Assessment of the average calculation time of the algorithms.

Algorithm	$\mathrm{Average}\;\mathrm{Calculation}\;\mathrm{Time}\;\mathrm{Per}\;\mathrm{Step}\;{\bar{t}}_{k}\;$
SR-UKF unconstrained	4,08 ms
SR-EKF unconstrained	4,27 ms
SR-UKF w. simplified Newton descent search	5,93 ms
SR-UKF w. sigma point projection constraints	5,19 ms

## Abbreviations

**Formula symbol**	**Unit**	**Description**
l	[−]	State of charge
h	[V]	Cell hysteresis voltage
$\;k_{i}\;$	[−]	MESC model cell capacity correction factor
$\;C_{N}\;$	[Ah]	Cell nominal capacity
$\;\eta_{Ah}\;$	[−]	Coulombic cell efficiency
h	[V]	Transient cell hysteresis voltage
M	[V]	Cell polarization voltage depend from 𝑙,𝑇

**Nomenclature**	**Explanation**
e	Lower case letter variable is a scalar
e	Bold lower case letter variable is a vector
E	Bold upper case letter variable is a matrix
$\begin{array}{c} e=\left\{e_{1},e_{2}\right\}\\ ={\left(e_{1},e_{2}\right)}^{T} \end{array}$	Equivalent vector notations
$\;E=\left[\begin{array}{cc} e_{11} & e_{12}\\ e_{21} & e_{22} \end{array}\right]\;$	Matrix notation
ϵ	Denotes the machine precision according to IEEE 754-2008

**Abbreviation**	**Explanation**
AUTOSAR	Automotive open system architecture
BMS	Battery management system
CAN	Controller area network—vehicle bus standard
DLR	German Aerospace Center
DLR SR	Insititute of System Dynamics and Control at DLR
Dymola	Modelica simulator from Dassault Systèmes
EMPHYSIS	Embedded systems with physical models in the production code software – ITEA 3 project
EKF	Extended Kalman filter
ESC	Enhanced self-correcting (model)
FIT	Normalized root mean square error criteria
FMI	Functional mockup interface
FMU	Functional mockup unit
FTP-75	(Environmental Protection Agency) Federal Test Procedure (drivecycle)
LAPACK	Linear Algebra PACKage–software library for numerical linear algebra
MATLAB	MATrix LABoratory–numerical computing software from MathWorks Inc.
MESC	Modified enhanced self-correcting (model)
MHE	Moving horizon estimation/estimator
Modelica	Object oriented modeling language for multiphysical systems [1]
MOPS	Multi-Objective Parameter Synthesis–DLR optimization software
MODRIO	Model Driven Physical Systems Operation – ITEA 3 project
NG	Nonlinear gradient (descent search)
NMPC	Nonlinear model predictive control
OCV	Open circuit voltage
PM	Perfect measurement
QP	Quadratic program
ROboMObil	see ROMO
ROMO	short for ROboMObil—DLR’s robotic electric vehicle
RTE	Run time environment
RTI	Real-time iteration
RTOS	Real-time operation system
SOC	State of charge [−] ∈[0,1]
SPT	Sigma point transformation
SQP	Sequential quadratic program
SR	Square-root
SR-EKF	Square-root extended Kalman filter
SR-UKF	Square-root unscented Kalman filter
UD-EKF	UD-decomposition based extended Kalman filter
UKF	Unscented Kalman filter
URNDDR	Unscented recursive nonlinear dynamic data reconciliation
XML	Extensible markup langua
Modelica	Object oriented modeling language for multiphysical systems [1]
MOPS	Multi-Objective Parameter Synthesis – DLR optimization software
NG	Nonlinear gradient (descent search)
NMPC	Nonlinear model predictive control
OCV	Open circuit voltage
PM	Perfect measurement
QP	Quadratic program
ROboMObil	see ROMO
ROMO	short for ROboMObil – DLR’s robotic electric vehicle
RTE	Run time environment
RTI	Real-time iteration
RTOS	Real-time operation system
SOC	State of charge [−] ∈[0,1]
SPT	Sigma point transformation
SQP	Sequential quadratic program
SR	Square-root
SR-EKF	Square-root extended Kalman filter
SR-UKF	Square-root unscented Kalman filter
UD-EKF	UD-decomposition based extended Kalman filter
UKF	Unscented Kalman filter
URNDDR	Unscented recursive nonlinear dynamic data reconciliation
XML	Extensible markup langua

## Appendix A. Cell Data Li-Tec HEI40

The lithium-ion cell under investigation is a pre-series production cell from Li-Tec Battery GmbH of the type ICS (Ion,Ceramic,Sheet Type) 12/203/245 (DIN IEC 61960). The main technical specificationas are given in the following:

**Characteristica**	**Value**	**Explanatory notes**
$\mathrm{Nominal}\;\mathrm{capacity}\;C_{N}\;$	40 [Ah]	
Charging end-voltage	4.2 [V]	
Nominal voltage	3.6 [V]	
Discharging end-voltage	3.0 [V]	
AC impedance	<0.7[mΩ]	
Gravimetric energy density	135 [Wh/kg]	
Volumetric energy density	241 [Wh/l]	
Electrolyte	n.a.	proprietary from Li-Tec Battery GmbH–see German patent DE102009040562
Seperator material	$\;{\mathrm{Al}}_{2}O_{3}{\mathrm{SiO}}_{2}\;$	ceramic separator – Evonic Separion©
Anode material	Graphite	Evonic Litarion©
Cathode material	${\;\mathrm{LiNi}}_{x}{\mathrm{Mn}}_{y}{\mathrm{Co}}_{z}O_{2}\;$	Evonic Litarion©
